# Current Practices and Preferences Regarding Race and Spirometry Interpretation: A National Survey

**DOI:** 10.1016/j.chpulm.2026.100239

**Published:** 2026-02-21

**Authors:** J. Henry Brems, Aparna Balasubramanian, Kadija Ferryman, Robert Wharton, Michelle N. Eakin, Meredith C. McCormack

**Affiliations:** a Division of Pulmonary and Critical Care Medicine, Johns Hopkins University, Baltimore, MD; b Berman Institute of Bioethics, Johns Hopkins University, Baltimore, MD

**Keywords:** PFTs, pulmonary function testing, race, reference equations, spirometry

## Abstract

**Background:**

The American Thoracic Society recently recommended the use of race-neutral rather than race-specific equations for spirometry interpretation. The uptake of and clinicians’ opinions about these practices remain unclear.

**Research Question:**

What are clinicians’ current practices and opinions around race-specific vs race-neutral reference equations?

**Study Design and Methods:**

We conducted a national survey of pulmonologists identified through the American Lung Association and Pulmonary Fibrosis Foundation. Participants completed an electronic survey about their opinions and practices related to race in spirometry reference equations. Descriptive and thematic analyses were conducted.

**Results:**

We received and analyzed 66 responses (29% response rate). Respondents had a median age of 48 years (interquartile range, 41-55), and 63 (95%) worked in an academic setting. A total of 34 respondents (54%) reported that their pulmonary function testing laboratory is already using a race-neutral equation. Most (n = 42, 64%) recommended that race-neutral equations should be used for spirometry interpretation, and 6 (9%) recommended race-specific equations alone should be used. When considering alternatives to race-specific equations, the use of Global Lung Function Initiative (GLI)-Global or GLI-Other as a single race-neutral equation was the most preferred alternative (n = 47, 71%). The most cited advantages of race-neutral equations were (1) improving equity, fairness, and bias (48%); and (2) improving accuracy and classification of disease (40%). The most cited disadvantages were (1) misclassification of respiratory disease (25%) and (2) loss of precision of lung function estimates (13%).

**Interpretation:**

Our results show that most academic pulmonologists would prefer to use a race-neutral rather than race-specific equation for spirometry interpretation. Although most have already transitioned to a race-neutral approach, significant gaps in implementing GLI-Global remain and indicate needs for ongoing research and education.

**Take-Home Points**

**Study** **Question:** What are clinicians’ current practices and preferences regarding the use of race-specific vs race-neutral equations for the interpretation of spirometry?

**Results:** Among 66 respondents, 54% reported that their pulmonary function testing laboratory is using a race-neutral equation, and a total of 42 (64%) recommend that race-neutral equations should be used for spirometry interpretation, with Global Lung Function Initiative (GLI)-Global or GLI-Other ranked as the most preferred alternative (n = 47, 71%) when transitioning away from race-specific equations.

**Interpretation:** Most clinicians surveyed support the use of a race-neutral approach for spirometry interpretation, but significant gaps in implementing GLI-Global remain.

An American Thoracic Society (ATS) statement recommends the use of a race-neutral strategy for the interpretation of pulmonary function testing (PFT).^1^ This recommendation was made in light of increasing evidence that a race-neutral approach—defined by not requiring race to apply a reference equation^1^—performs as well or better than race-specific equations for predicting disease and mortality.^2^, ^3^, ^4^ Furthermore, the use of race in PFT interpretation can promote incorrect and harmful beliefs about a biological basis to race.^1^^,^^5^ In addition, the transition to race-neutral PFT interpretation may result in improved access for Black patients to disease-specific treatments, lung transplantation, and disability benefits.^6^, ^7^, ^8^

Despite the updated recommendation, it remains unknown to what degree clinicians and PFT laboratories have shifted to using a race-neutral reference equation. Understanding the implementation of Global Lung Function Initiative (GLI)-Global—the recommended race-neutral reference equation—is important for at least 2 reasons. First, as previously noted, it may promote racially equitable care. Second, standardization of reference equations across PFT laboratories can aid the transferability of care and applicability of guidelines between medical centers.^9^

Pulmonologists and clinicians who frequently interpret PFTs are key stakeholders in the process of transitioning to a race-neutral approach—both because they are likely to influence PFT laboratories’ choice of reference equations and because they are end-users of PFTs who apply the results in a clinical setting. However, little is known about their understanding of or opinions about a potential transition to a race-neutral approach. For example, it is unclear to what extent clinicians agree with the ATS recommendation to use a race-neutral approach in general or to use GLI-Global specifically.^10^^,^^11^ Additionally, it is unknown how well clinicians understand the impact of a shift to a race-neutral approach, despite its potential to alter clinical and nonclinical (eg, employment, disability benefits) decision-making.^1^^,^^7^^,^^12^ Thus, to support the transition to GLI-Global, further data are needed about the current practices and preferences related to the use of race in spirometry interpretation.

In this study, we sought to assess clinicians’ attitudes toward a race-neutral equation, including their opinion on the use of race in spirometry interpretation in general and the favorability of GLI-Global specifically, among a national sample of pulmonologists.

## Study Design and Methods

### Survey Design

The survey instrument for this study was developed by the investigators. An initial set of questions was adapted from a prior analogous study investigating opinions on use of race in calculating glomerular filtration rate (GFR), which investigated concepts related to current institutional practices and opinions regarding use of race, including (1) whether race should be used in GFR estimation, (2) perceived benefits and harms of removing race, and (3) alternative methods for GFR calculation.^13^ To establish face and content validity, the instrument was iteratively updated and piloted with members of the ATS Pulmonary Function Testing Committee and with pulmonary fellows from Johns Hopkins University.

The final instrument included questions on participant characteristics, attitudes toward reference equations, current spirometry interpretation practices, and awareness of the impact about race-neutral equations. To evaluate attitudes, we assessed participants’ recommendations regarding the use of race in spirometry interpretation, the favorability of GLI-Global and other alternatives to race-specific interpretation, and the perceived benefits and harms of eliminating race-specific interpretation. Current practices were assessed to understand what sets of equations (eg, GLI, National Health and Nutrition Examination Survey) are currently in use and to what extent race-neutral equations are already used. We assessed awareness of the impact of reference equations on percent predicted values by race because a shift from race-specific to race-neutral equations would produce an artificial change in percent predicted values, which has the potential to alter clinical interpretation and care. Overall, these concepts were selected given ongoing debate related to these topics and the recognized uncertainty around these issues in the statement recommending a race-neutral approach.^1^^,^^10^^,^^11^^,^^14^ Because a major goal was to assess attitudes toward race-neutral equations, our coprimary outcomes were respondents’ (1) recommendations regarding the use of race in spirometry interpretation and (2) the favorability of GLI-Global.

The full survey instrument is available in e-Appendix 1. This study was approved by the Johns Hopkins School of Medicine institutional review board (No. IRB00393882).

### Survey Administration

Potential participants were recruited through partnerships with the American Lung Association and the Pulmonary Fibrosis Foundation (PFF), who provided contact information for affiliated clinicians. Eligible participants included those with an MD or equivalent degree who completed medical training and are actively engaged in patient care. All participants were invited via email to complete the survey electronically via REDCap. Two reminder emails were sent out at 1-week intervals. The survey was administered from February 26, 2024, to May 6, 2024.

### Statistical Analysis

Descriptive statistics were generated for all variables using median (interquartile range) for continuous data and frequency (%) for categorical data. For all questions, we calculated percentages as the number of responses divided by the total number of survey respondents, whether or not all respondents answered that question. For questions which included an option of not sure or unknown, missing responses were considered as a response for not sure or unknown. Otherwise, missing responses were considered as their own category for each question to allow for their inclusion in all analyses.

To assess the favorability of alternative options to race-specific reference equations, we initially reported the 5-point Likert scale as continuous data (median [interquartile range]). Using the Wilcoxon signed rank test, we then compared favorability of all options with the favorability of GLI-Global or GLI-Other because this option most closely reflects the current recommendations for spirometry recommendation.^1^ We subsequently evaluated the proportion of respondents who considered each potential alternative as favorable, defined as a ranking of 4 or 5 on the 5-point scale.

To explore the association between respondent characteristics and an opinion that race-neutral equations should be used for spirometry interpretation, we used Firth’s penalized logistic regression to account for the sample size and number of characteristics assessed. An opinion to use race-neutral equations was defined as a binary variable according to whether respondents explicitly selected that option when asked about their general recommendation. Covariates included age, race, gender, frequency of PFT interpretation, a correct answer to at least 1 of 2 questions about the impact of race-specific and race-neutral equations on percent predicted values, and whether the respondent’s PFT laboratory had already implemented a race-neutral equation.

Free-text responses to 2 questions regarding the perceived single greatest advantage and disadvantage of race-neutral equations were thematically analyzed using an inductive approach by 2 authors (J. H. B. and R. W.). Both authors independently analyzed all responses and resolved all discrepancies via discussion.

A 2-sided *P* value < .05 was considered statistically significant. All statistical analyses were performed using STATA version 17.0 (StataCorp).

## Results

A total of 231 surveys were delivered to email addresses provided by the American Lung Association and PFF. Among the 231 individuals who received the survey, there were 66 responses for an overall response rate of 29%. Of the 66 responses, 6 included missing responses to at least 1 question.

Respondent characteristics are summarized in Table 1. Overall, most respondents identified as men (61%), were White participants (64%), and worked in an academic setting (95%). Twenty-five states were represented among all respondents. There were 64 respondents (97%) who practiced as pulmonologists, and a total of 58 (88%) interpreted PFTs at least weekly or more often. Although most respondents (91%) interpreted PFTs in the clinical care of their patients, 35 (53%) also interpreted for a PFT laboratory and 8 (12%) directing a PFT laboratory.

**Table 1:** Characteristics of Survey Respondents (N = 66)

Characteristic	Value
Age, y	48 (41-55)
Gender	
Man	39 (59)
Woman	26 (39)
Ethnicity	
Hispanic	3 (5)
Non-Hispanic	59 (89)
Race	
Asian	19 (29)
Black	1 (2)
White	42 (64)
Other	4 (6)
Region^a^	
Western	8 (12)
Central	20 (30)
Southern	15 (23)
Northeast	16 (24)
Years since completing training	
0-10	24 (36)
11-20	20 (30)
21-30	14 (21)
> 30	8 (12)
Primary work setting	
Academic	63 (95)
Community	1 (2)
Government	1 (2)
Frequency of PFT interpretation	
Daily	20 (30)
Multiple days per week	20 (30)
Weekly	18 (27)
Monthly	4 (6)
Less than monthly	1 (2)
Role(s) in which PFTs are interpreted^b^	
Clinical care of own patients	60 (91)
Interpreting for PFT laboratory	35 (53)
Directing PFT laboratory	8 (12)
Teaching	14 (21)
Research	7 (11)

Data are presented as either median (25th-75th percentile) or No. (%). PFT = pulmonary function test.

a Regions were defined by state according to American Association of Medical Colleges classification.^15^

b Participants were allowed to select > 1 option.

### Attitudes

**Figure 1:**
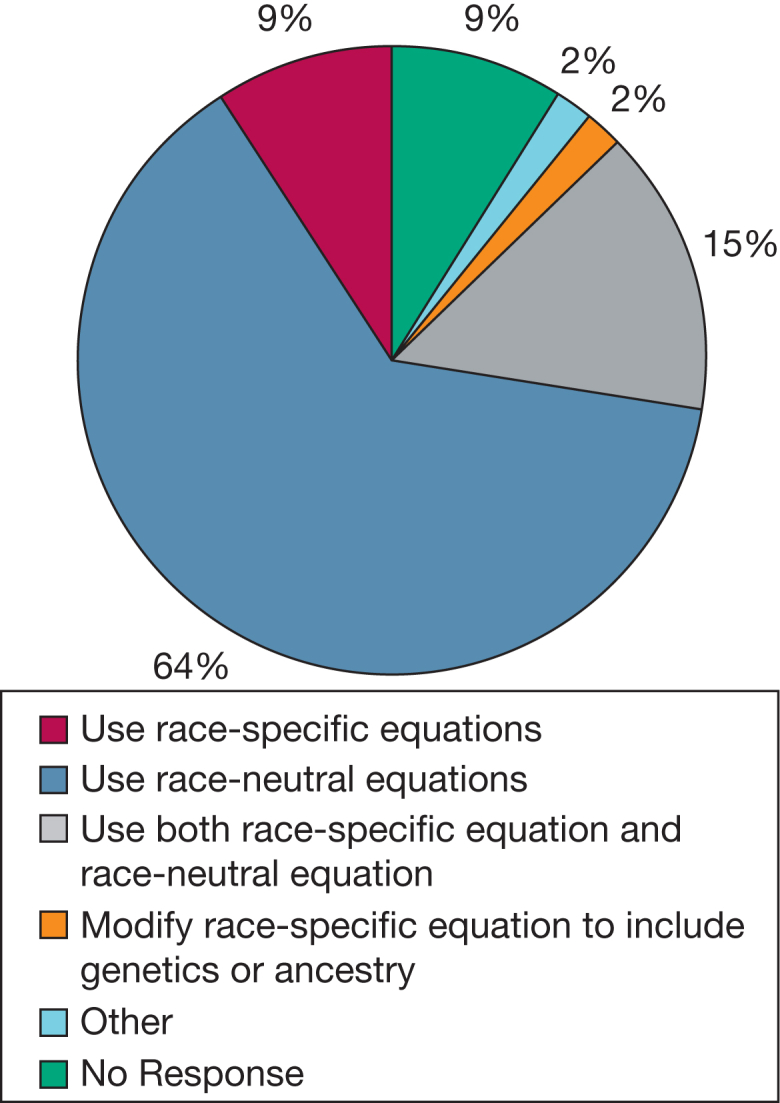
Opinions regarding use of race in reference equations. The percent of respondents selecting each option in response to the following question: What is your general recommendation regarding the use of race-specific reference equations for the interpretation of spirometry (eg, the calculation of percent predicted values)? The respondent selecting other indicated a preference to “focus on lower limits of normal (LLNs) rather than % predicted.”

Respondents’ opinions about the use of race in spirometry interpretation are displayed in Figure 1. Most respondents (64%) indicated a preference to use race-neutral reference equations for spirometry interpretation. Only 6 respondents (9%) preferred to continue to use race-specific equations instead.

**Figure 2:**
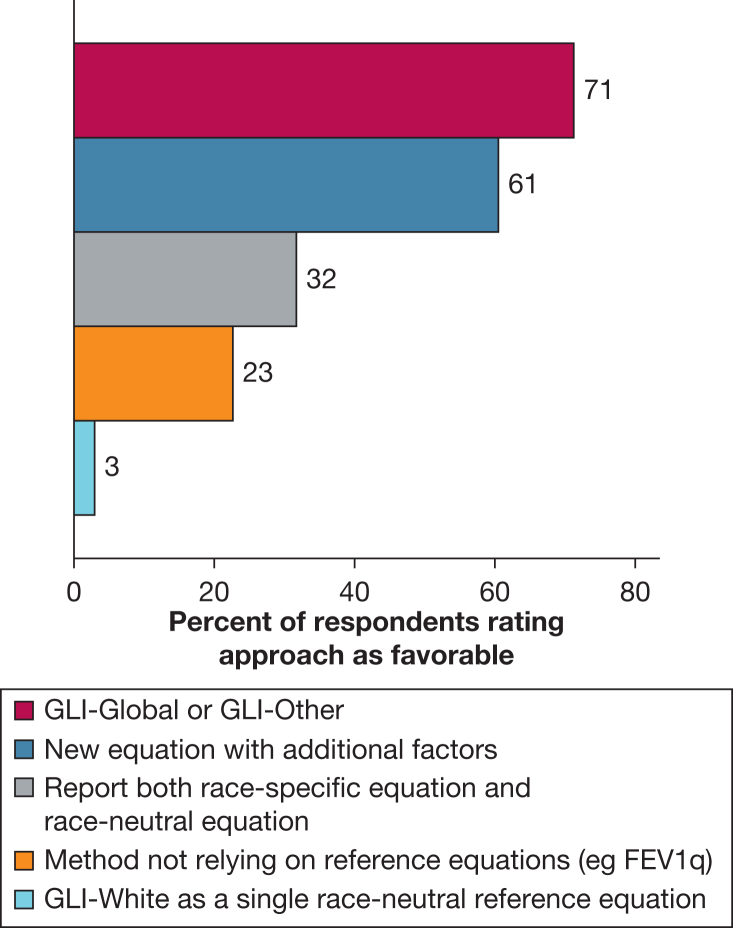
Favorability of alternative approaches to race-specific equations for spirometry interpretation. The percent of respondents who rated each option as a 4 or 5 on a 5-point Likert scale when asked the following: Please rank the following options on how favorable you think they would be as potential alternatives to race-specific equations. FEV1q = FEV1 Quotient; GLI = Global Lung Function Initiative.

When asked to consider potential alternatives to race-specific equations, 47 respondents (71%) considered the use of GLI-Global or GLI-Other as a single race-neutral equation to be somewhat or very favorable. Only 3 respondents (5%) considered it unfavorable. The favorability of all alternatives is shown in Figure 2. When comparing favorability ratings on a 5-point Likert scale (where 1 is very unfavorable and 5 is very favorable), use of GLI-Global or GLI-Other as a single race-neutral equation was considered more favorable than all other alternatives except for use of a potential new equation that would incorporate additional factors (median Likert scale rating, 4 vs 4; *P* = .71) (Table 2).

**Table 2:** Comparison of Favorability of Alternative Interpretive Approaches to Race-Specific Reference Equations

Survey Question	Favorability, Median (IQR)	*P* Value^a^
Please rank the following options on how favorable you think they would be as potential alternatives to race-specific equations (1 = very unfavorable, 5 = very favorable)		
GLI-Other or GLI-Global as a single race-neutral reference equation	4 (4-4)	ref
GLI-White as a single race-neutral reference equation	2 (1-3)	< .001
A new race-neutral reference equation that incorporates other patient factors (eg, seated height)	4 (3-4)	.71
Offering percent predicted values from both race-specific and race-neutral equations	3 (2-4)	< .001
Using a method that does not rely on reference equations (eg, FEV_1_q)	3 (2-3)	< .001

FEV1q = FEV1 Quotient; GLI = Global Lung Function Initiative; IQR = interquartile range; ref = reference.

a *P* value calculated using Wilcoxon signed-rank test for all options individually compared with GLI-Global or GLI-Other as reference.

In the multivariable regression analysis, prior implementation of a race-neutral equation at one’s own PFT laboratory was the only variable significantly associated with an opinion to use race-neutral equations (OR, 5.41; *P* = .009). The model demonstrated adequate fit using the Hosmer-Lemeshow test (*P* = .87), and full results are displayed in e-Table 1.

In thematic analyses, the 2 most cited advantages of race-neutral reference equations were that they (1) improve equity, fairness, or bias (48%); or (2) improve accuracy and classification of spirometry (40%). The 2 most cited disadvantages of a transition to race-neutral interpretation were concerns regarding (1) misclassification of respiratory disease (25%) and (2) loss of precision in lung function estimates (13%). Examples of responses citing these advantages and disadvantages are displayed in Table 3.

**Table 3:** Examples of Respondent Quotes to the Most Commonly Cited Advantages and Disadvantages to Using Race-Neutral Reference Equations

Advantages or Disadvantages	Respondent Quotes
Advantages	
Improves equity, fairness, or bias	“It may reduce health disparities caused from by underestimating disease severity in racial minority groups.”
	“Race is a social construct, so we are avoiding unproven historical concepts when providing care to patients.”
	“Since spiro often used to determine eligibility for more advanced pulmonary therapies (transplant/LVRS) it will help improve access to all patients.”
Improves accuracy and classification of disease	“Improves detection of lung disease among minority populations.”
	“Race-neutral reference equations provide improved relationships between the values on spirometry, symptoms, and functional capacity and also allow for identification of modifiable risk factors that may otherwise be missed in certain individuals.”
	“People of color aren't read as normal PFTs when they are abnormally low.”
Disadvantage	
Misclassification of respiratory disease	“There are differences that exist that don't appear to be entirely explained by socio-economic differences etc and thus we may risk calling some 'normal' patients abnormal by ignoring these differences.”
	“May reclassify some patients as no longer having 'disease' who potentially do and/or rely on that diagnosis for aspects of health care and reimbursement.”
Loss of precision in lung function estimates	“Elimination of factors that are causally independent but correlated with race and which are important for determining lung function.”
	“Offers a one size fits all approach where precision markers May have added advantage. They additionally still incorporate race in their derivation which presents residual bias.”
Difficulty in comparing spirometry values over time	“Changing from specific to neutral can confuse both patients and doctors who are following PFTs.”
	“Impacts longitudinal messaging when comparing to race-adjusted % predicted.”

LVRS = Lung volume reduction surgery; PFT = pulmonary function testing.

### Current Practices

Among all survey respondents, 47 (71%) reported that their PFT laboratory uses a GLI equation, 12 (18%) reported that their laboratory uses National Health and Nutrition Examination Survey equations, and 7 (11%) were unsure of which equation was being used (e-Table 2).

A total of 34 respondents (54%) noted that their PFT laboratory is already using a race-neutral equation. Nineteen respondents (30%) reported using a race-specific equation, whereas 10 (16%) were not sure. Among the 34 respondents reporting use of a race-neutral equation, 76% of those reported which specific equation was being used, and in all cases, a GLI equation (GLI-Global or GLI-Other) was the race-neutral equation implemented (e-Table 2).

### Awareness of Impact

When asked how the FEV_1_ % predicted would compare between an otherwise matched (ie, same age, sex, and height) White and Black patient when using either race-specific or race-neutral equations, 38 of the 63 respondents (60%) answered correctly when considering race-specific equations, and 81% of respondents answered correctly when considering a race-neutral equation (e-Fig 1). Among the 63 respondents to both questions, a total of 57 (90%) answered at least 1 correctly, whereas 32 (51%) answered both questions correctly.

Results of responses to other questions in the survey instrument, including about the perceived importance of percent predicted values (e-Table 3) and the use of sex in reference equations (e-Table 4), are detailed in e-Appendix 1.

## Discussion

In this national survey of predominantly academic pulmonologists, we found that most respondents support and are using race-neutral equations for spirometry interpretation. These findings have important implications in light of ongoing efforts to deimplement the use of race in clinical algorithms.^16^

Only a very small minority of clinicians surveyed in our study would favor continued use of race-specific equations alone, and among alternatives to a race-specific approach, use of the currently recommended GLI-Global or GLI-Other equation appears to be the most acceptable.^1^ In addition, our finding that prior implementation of a race-neutral equation is strongly associated with an opinion in favor of race-neutral equations suggests that clinicians may be likely to ultimately find a transition to GLI-Global favorable. Together, these findings support the acceptability of ongoing efforts to transition to race-neutral equations.

Our study builds on prior data and demonstrates the evolution of clinicians’ opinions on the use of race in spirometry. In a study conducted in 2021 before the ATS recommendation to use race-neutral equations, most clinicians had favored using race-specific equations compared with only 9% recommending such an approach in our study.^14^ In a more recent study of 47 division directors conducted 6 months after the ATS recommendation, 66% of institutions had transitioned to a race-neutral interpretation strategy, which roughly aligns with our own findings.^17^ More broadly, the adoption of a race-neutral interpretation of pulmonary function somewhat parallels that of GFR, which saw a rapid increase from 30% of laboratories using the race-neutral Chronic Kidney Disease Epidemiology Collaboration equation in 2022 to 66% in 2023.^18^, ^19^, ^20^

The practices and opinions of other clinicians, including those working in community or government practices, remain important and underrepresented. We predominantly sampled clinicians from academic centers, who may be more likely than physicians at nonacademic centers to be aware of and to adopt recommendations to use race-neutral equation, as has been the case for GFR equations.^19^ Important differences also likely exist among those practicing within the Department of Veterans Affairs, which had reported that approximately 30% of laboratories had switched to a race-neutral approach before pausing the transition.^21^ Future research among these populations is warranted to understand the implementation of race-neutral equations.

Our findings highlight other important areas for future research. The second-most favored alternative to race-specific equations in our study was a new (race-neutral) equation that incorporates additional patient factors, which—although hypothetical—may indicate interest in further research related to anthropometric or genetic factors related to lung function.^22^, ^23^, ^24^, ^25^, ^26^ Many respondents to our survey also noted concerns that race-neutral equations could lead to misclassification of disease and a loss of precision in estimating lung function. Although increasing data support the accuracy of race-neutral reference equations for classifying respiratory disease,^2^, ^3^, ^4^^,^^27^^,^^28^ further research may assuage concerns and remains necessary given the broad clinical and nonclinical implications of references equations.^7^^,^^29^

Educational initiatives may also be helpful as PFT laboratories transition to race-neutral reference equations. Our survey instrument did not provide a validated assessment of respondents’ knowledge, but the significant proportion of respondents in our survey who incorrectly answered questions related to the impact of race-specific and race-neutral equations on percent predicted values may reflect how easy it is for clinicians to misinterpret spirometry when transitioning equations. In addition, respondents did not commonly cite concerns around eligibility for lung cancer resection or disability benefits when considering a transition to race-neutral equations.^12^^,^^29^ Although this point does not necessarily indicate respondents were unaware of these impacts, it may underscore a need for further education for clinicians about scenarios in which a transition to race-neutral equations most clearly impact delivery of care.

The response rate of 29% is a key limitation of our study and may indicate potential nonresponse bias. Although characteristics of nonrespondents are unknown, prior data indicate broadly similar distributions by race in our study (64% White, 29% Asian, and 2% Black participants) as among clinicians in the PFF clinical care network (67% White, 20% Asian, and 3% Black participants); however, there were fewer participants in our study who had been in practice for < 10 years (36% vs 50%).^30^

Multiple other strengths and limitations of our study warrant consideration. We primarily surveyed academic pulmonologists, and our results cannot be extrapolated to all pulmonologists or PFT laboratories. It is possible that a large proportion of clinicians other than those surveyed here—particularly including primary care physicians, surgeons, and/or those not practicing at academic centers—may be less likely to have adopted a race-neutral approach. Nonetheless, to our knowledge this is the largest study to evaluate practices and preferences related to race and reference equations since the publication of the ATS recommendation to use a race-neutral approach, and we surveyed a national sample of clinicians with broad geographic distribution. In addition, our study did not assess racial makeup of respondents’ PFT laboratories, which could influence uptake of GLI-Global. Our study was also likely underpowered to thoroughly evaluate characteristics associated with a favorable opinion of race-neutral equations. Future research efforts are needed to understand national patterns and to formulate strategies to increase uptake of GLI-Global.

## Interpretation

We found that most academic pulmonologists would prefer to use a race-neutral rather than race-specific equation for spirometry interpretation. Although most have transitioned to a race-neutral approach already, significant gaps in implementing GLI-Global remain and indicate needs for ongoing research and education.

## Funding/Support

The research reported in this publication was supported by NHLBI of the NIH [Awards F32HL165771 [J. H. B.], T32HL007534 [J. H. B.]). J. H. B is a K12 scholar funded by K12TR004925.

## Financial/Nonfinancial Disclosures

None declared.

## Supplementary Data

e-Online Data
