# Supplementary material for: Current Practices and Preferences Regarding Race and Spirometry Interpretation: A National Survey
Source: CHEST Pulm. 2026 Feb 21;4(2):100239. doi: 10.1016/j.chpulm.2026.100239 (PMC13418350; doi:10.1016/j.chpulm.2026.100239)
Supplement: e-Online Data [file mmc1.docx]

**Online Supplement**

**Current Practices and Preferences regarding Race and Spirometry Interpretation: A National Survey**

**e-Table 1.** Results of logistic regression models for association between respondent characteristics and a recommendation to use race-neutral reference equation for spirometry interpretation.

| **Coefficient** | **Odds Ratio (95% CI)** | **P value** |
| --- | --- | --- |
| Age (years) | 1.00 (0.94 – 1.06) | 0.99 |
| Gender |  |  |
| Male | *ref* |  |
| Female | 1.27 (0.37 – 4.38) | 0.70 |
| Race and Ethnicity^a^ |  |  |
| Asian | *ref* |  |
| White | 3.55 (0.97 – 13.0) | 0.06 |
| Underrepresented Minorities | 0.90 (0.12 – 6.5) | 0.92 |
| Frequency of PFT interpretation |  |  |
| Less than weekly | *ref* |  |
| Weekly or more often | 1.32 (0.21 – 8.36) | 0.82 |
| Answers to question about impact of reference equations on %-predicted values^a^ |  |  |
| Incorrect response to both questions | *ref* |  |
| Correct response to at least one question | 4.42 (0.74 – 26.5) | 0.10 |
| Implementation of race-neutral equation at own PFT lab |  |  |
| Not yet implemented | *Ref* |  |
| Already implemented | 4.90 (1.40 – 17.2) | 0.01 |

a. Underrepresented minorities included any individual who reported “Black” or “Other” race or who reported “Hispanic” ethnicity

b. Defined by answer to question: “A Black and a White patient are the same age, sex, and height. Both have a measured FEV1 of 2.0L. How will their FEV1 percent-predicted compare when calculated using the following:” for a scenario using either race-specific or race-neutral equation.

**e-Table 2**. Currently utilized reference equations for spirometry interpretation.

| **Survey Question** | **Responses, n (%)**  **(N = 66)** |
| --- | --- |
| Which reference equations does your PFT lab use for spirometry? |  |
| GLI | 47 (71%) |
| NHANES | 12 (18%) |
| Not sure^a^ | 7 (11%) |
| Has your PFT lab implemented a method for using a race-neutral (i.e. non-'race-specific') reference equation? |  |
| Yes | 34 (52%) |
| No | 19 (29%) |
| Not sure^a^ | 13 (20%) |
| What race-neutral reference equation has your lab implemented? |  |
| GLI (Global or Other) | 26 (76%) |
| Unknown^a^ | 8 (24%) |

a Missing responses included as “Not Sure” or “Unknown”

**e-Table 3**. Perceived importance of percent predicted spirometry values

|  | **Favorability**  **(median, IQR)** |
| --- | --- |
| **On a scale of 1-5, how would you rate the importance of percent predicted values when assessing a patient’s FEV1 and FVC in the following scenarios:** (1 = Not at all important, 5 = Extremely important) |  |
| Evaluation of a new patient with a respiratory complaint, such as dyspnea | 5 (4 – 5) |
| Monitoring the lung function of a patient with chronic respiratory disease over time | 4 (4 – 5) |

**e-Table 4**. Opinions regarding the use of sex in reference equations

| **Survey Question** | **Responses, n (%)**  **(N = 66)** |
| --- | --- |
| What is your opinion on the use of sex in spirometry reference equations? |  |
| Sex-specific equations are necessary and should be used in estimating lung function | 24 (36%) |
| Sex-specific equations should NOT be used in estimating lung function | 2 (3%) |
| Sex-specific equations should be generally applied for cis-gender patients, but an alternative approach is needed for trans-gender and non-binary patients | 19 (29%) |
| Unsure, or more research needed to determine if sex-specific equations should be used^a^ | 21 (32%) |

a A total of 5 missing responses were included as “Unsure”

**
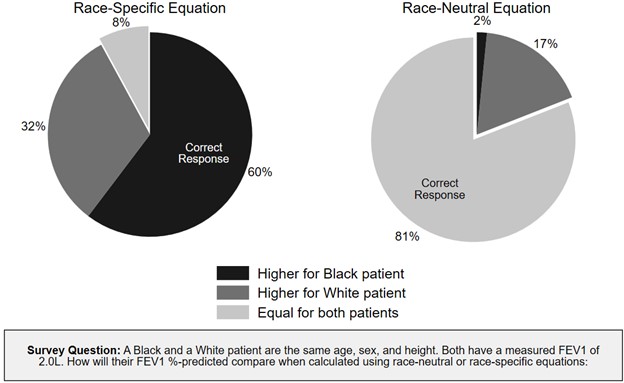
**

**e-Figure 1**. Respondents’ understanding of how reference equations impact %-predicted values according to race. Percentages of all responses to the question “A Black and a White patient are the same age, sex, and height. Both have a measured FEV1 of 2.0L. How will their FEV1 percent-predicted compare when calculated using the following:” are shown below. Respondents were provided the same prompt and answer choices under a scenario of using race-specific (left) or race-neutral (right) reference equations.

**Survey Instrument**

| **Survey Questions** | **Response Format** | **Response Options** |
| --- | --- | --- |
| 1.) Which of the following degrees or certifications do you hold?  [End if not MD, DO, equivalent] | Select all that apply | - M.D., D.O., or equivalent (e.g. M.B.B.S.) - PhD - PA or CRNP - RN - CRT or CRTT - Master’s degree - Other clinical degree - Other non-clinical degree |
| 2.) How many years has it been since you have completed training?  [End if in residency] | Single answer | - Have not yet completed training -- in residency - Have not yet completed training – in fellowship - 0-10 - 11-20 - 21-30 - More than 30 years |
| 3.) In which of the following settings do you see patients?  [End if neither] |  | - Outpatient - Inpatient - Both - Neither |
| 4.) What is your primary work setting? | Single answer | - Academic - Community - Government - Industry (e.g. pharmaceutical) - Other (please specify) |
| 5.) Which describes your practice or type of work? | Single answer | - Pulmonology only - Critical Care only - Pulmonary and Critical Care - Internal Medicine only - Other (please specify) |
| 6.) What state do you primarily practice in? | Single answer | - [list of states] |
| 7.) What is your age? | Single answer | - [number entry] |
| 8.) What gender do you identify with? | Single answer | - Male - Female - Non-binary |
| 9.) What is your ethnicity | Single answer | - Hispanic - Non-Hispanic |
| 10.) What is your race? | Select all that apply | - American Indian or Alaskan Native - Asian - Black or African American - Native Hawaiian or other Pacific Islander - White - Other (please- specify) |
| 11.) How often do you interpret a patient’s PFT report? | Single answer | - Daily - Multiple days per week - Weekly - Monthly - Less than monthly |
| 12.) In which of the following roles do you apply your knowledge of PFTs? | Select all that apply | - Clinical care of my own patients - Clinical care of patients other than my own (e.g. interpreting PFT reports for a PFT laboratory) - Directing a PFT lab - Teaching trainees via a formal course - Other (please specify) |
| 13.) On a scale of 1-5 (1 = Not at all important, 5 = Extremely important), how would you rate the importance of percent predicted values when assessing a patient’s FEV1 and FVC in the following scenarios:  Evaluation of a new patient with a respiratory complaint, such as dyspnea? | Single answer | - 1-5 |
| 14) On a scale of 1-5 (1 = Not at all important, 5 = Extremely important), how would you rate the importance of percent predicted values when assessing a patient’s FEV1 and FVC in the following scenarios:  Monitoring the lung function of a patient with chronic respiratory disease over time? | Single answer | - 1-5 |
| 15.) A Black and a White patient are the same age, sex, and height. Both have a measured FEV1 of 2.0L. How will their FEV1 percent-predicted compare when calculated using race-specific reference equations (i.e. Global Lung Function Initiative [GLI] ‘Black’ and ‘White’ equations used according to each patient’s race)? | Single answer | - Black patient will have higher FEV1% predicted - White patient will have higher FEV1% predicted - FEV1% predicted will be equivalent for both patients |
| 16.) A Black and a White patient are the same age, sex, and height. Both have a measured FEV1 of 2.0L. How will their FEV1 percent-predicted compare when calculated using race-neutral reference equations (i.e. same GLI equation applied to each regardless of race, such as GLI-Global)? | Single answer | - Black patient will have higher FEV1% predicted - White patient will have higher FEV1% predicted - FEV1% predicted will be equivalent for both patients |
| 17.) Which reference equations (whether race-specific or race-neutral) does your PFT lab use for spirometry? | Single answer | - GLI - NHANES (Hankinson) - Morris, Crapo, or Knudson - Equations derived from a local cohort - Not sure - Other (please specify) |
| 18.) Has your PFT lab implemented a method for using a race-neutral (i.e. non-‘race-specific’) reference equation? | Single answer | - Yes - No - Not sure |
| [If yes to 18]  19.) What race-neutral reference equation has your lab implemented | Single answer | - [Free response] |
| 20.) What is your general recommendation regarding the use of race-specific reference equations for the interpretation of spirometry (e.g. the calculation of percent predicted values)? | Single answer | - Use race-specific equations (e.g. GLI or NHANES) - Use ‘race-neutral’ equations (e.g. GLI ‘Global’ or ‘Other’) - Use both race-specific and race-neutral equations, and report values from both - Modify race-specific equations to include genetics or ancestry - Other (please specify) |
| 21-25) Please rank the following options on how favorable you think they would be as potential alternatives to race-specific equations (1 = Not at all favorable, 10 = Extremely favorable) | Single answer for each (1-5) | - GLI ‘Other’ or GLI ‘Global’ as a single “race-neutral” reference equation - GLI ‘White’ as a single “race-neutral” reference equation - A new “race-neutral” reference equation that incorporates other patient factors (e.g. seated height) - Ancestry-specific equations - Offering percent predicted values from both race-specific and race-neutral equations on PFT reports - Other option (please specify below) |
| 26.) What is your opinion on the use of sex in spirometry reference equations? | Single answer | - Sex-specific equations are necessary and should be used in estimating lung function - Sex-specific equations should NOT be used in estimating lung function - Sex-specific equations should be generally applied for cis-gender patients, but an alternative approach is needed for trans-gender and non-binary patients - Unsure, or more research needed to determine if sex-specific equations should be used |
| 27.) What is the number one advantage of using race-neutral instead of race-specific equations? | Free response |  |
| 28.) What is the number one disadvantage of using race-neutral instead of race-specific equations? | Free response |  |
